# Classification of Horse Gaits Using FCM-Based Neuro-Fuzzy Classifier from the Transformed Data Information of Inertial Sensor

**DOI:** 10.3390/s16050664

**Published:** 2016-05-10

**Authors:** Jae-Neung Lee, Myung-Won Lee, Yeong-Hyeon Byeon, Won-Sik Lee, Keun-Chang Kwak

**Affiliations:** 1 Department of Control and Instrumentation Engineering, Chosun University, 375 Seosuk-dong, Gwangju 501-759, Korea; ljn1321@daum.net (J.-N.L.); mailsanai@daum.net (M.-W.L.); qasdfghjt@daum.net (Y.-H.B.); 2 Yudo-Star Co., ltd. 415, Cheongneung-Daero, Namdong-Gu, Incheon 405-817, Korea; wslee@yudostar.com

**Keywords:** classification of horse gaits, neuro-fuzzy classifier, fuzzy c-means clustering, inertial sensor, horse riding coaching

## Abstract

In this study, we classify four horse gaits (walk, sitting trot, rising trot, canter) of three breeds of horse (Jeju, Warmblood, and Thoroughbred) using a neuro-fuzzy classifier (NFC) of the Takagi-Sugeno-Kang (TSK) type from data information transformed by a wavelet packet (WP). The design of the NFC is accomplished by using a fuzzy c-means (FCM) clustering algorithm that can solve the problem of dimensionality increase due to the flexible scatter partitioning. For this purpose, we use the rider’s hip motion from the sensor information collected by inertial sensors as feature data for the classification of a horse’s gaits. Furthermore, we develop a coaching system under both real horse riding and simulator environments and propose a method for analyzing the rider’s motion. Using the results of the analysis, the rider can be coached in the correct motion corresponding to the classified gait. To construct a motion database, the data collected from 16 inertial sensors attached to a motion capture suit worn by one of the country’s top-level horse riding experts were used. Experiments using the original motion data and the transformed motion data were conducted to evaluate the classification performance using various classifiers. The experimental results revealed that the presented FCM-NFC showed a better accuracy performance (97.5%) than a neural network classifier (NNC), naive Bayesian classifier (NBC), and radial basis function network classifier (RBFNC) for the transformed motion data.

## 1. Introduction

The grades for quality of life in the Republic of Korea, Japan, Canada, and the US are 5.8, 5.9, 7.3, and 7.2, respectively, according to the National Statistical Office (NSO)’s report of 2015. This indicates that the quality of life in South Korea is low, as compared to other countries. To resolve this issue, the objective of the present study is to facilitate the introduction of horse riding in Korea, which would contribute to improving the quality of life through communication and sport. Horse riding involves all the movements of walking and running on horseback. It is known to be a gentlemanly sport that promotes an individual’s bodily balance, flexibility, and courage while on horseback. The sport constitutes keeping in step with a living creature, not a machine. Therefore, it is important to pay special attention to safety and the mutual balancing of the horse and rider. Indeed, although it is well known that “riding horses is good” few learn how to ride a horse. This sport has a good influence on posture, bodily growth and the shape of the body, and emotional stability. In addition, the beneficial effects on weight management and the prevention of spinal disk damage have been highlighted. Reciprocal communication plays a significant role in horse riding, unlike other sports. Horse riding is considered to be of such high quality that it is called “a noble sport.” However, horse riding also requires coaching. The expensive coaching requirement makes the sport much more difficult for ordinary people to approach. However, unmanned coaching could reduce the financial burden and eventually help improve people’s life quality. The present study is designed to allow unmanned coaching through classifying horse gaits and recognizing the rider’s postures for calibration. 

Various studies have been conducted using inertial sensors, and wireless networking is suitable for effective horse riding coaching. Domestic studies on the effects of inertial sensors on the balance improvement of the elderly with dementia exist [1], as well as on reducing body weight [2], the proliferation of vascular smooth muscle cells [3], *etc.* Abroad, Luinge [4] proposed a precise method to measure human size using inertial and gyro sensors. Zhou [5] estimated human movements using inertial sensors. Lee [6] proposed a calibration method and sensor fusion for motion capture using an acceleration meter. Zhu [7] traced real-time movements. Venkatraman [8] investigated animal movements using a pattern recognition algorithm and neural network. Ghasemzadeh [9] proposed a golf swing training system. Mariani [10] assessed the walking of young and senior citizens. Jung [11] proposed a method for tracking upper body motions using inertial sensors. Song [12] proposed a practical calibration method of the MEMS (Micro Electro Mechanical Systems) gyroscope sensor. Wei [13] investigated MEMS calibration. Cao [14] examined the full swing in golf. Pyeong Gook [15] addressed smart shoes based on inertial sensors. Chan [16] developed a dancing training system for coaching. In the area of sports analysis and coaching using inertial sensors, a human motion acquisition system based on inertial sensors was implemented for self-coaching [17], a golf coaching system using human motion analysis was developed [18], a four joint-based motion capture system was studied for spinal disease protection [19], kinematic coaching analysis using wireless inertial sensors was proposed [20], tennis strokes were classified [21], and a golf training system was developed [22]. Although several studies have been conducted on using inertial sensors in various application areas [23,24,25,26,27,28,29], the research on automatic horse riding coaching through movement classification has not thus far been studied.

In this paper, we present a method for designing a fuzzy c-means (FCM)-based neuro-fuzzy classifier (NFC) of the Takagi-Sugeno-Kang (TSK) type for the classification of four gaits (walk, sitting trot, rising trot, and canter) in three breeds of horse (Jeju, Warmblood, Thoroughbred). FCM clustering performs fuzzy partitioning such that a given data point can belong to several groups, with the degree of belongingness specified by membership grades between 0 and 1. This clustering is used in conjunction with a neuro-fuzzy classifier primarily to obtain knowledge of automatic fuzzy if-then rules. Here, the sensor data information obtained by the motion capture system in this study is transformed by a wavelet packet (WP) to provide dimensional reduction. Using a capture system, which includes two channel receivers, as well as a PC and server, a database of riders’ motions was constructed using the data collected from a motion capture suit to which inertial sensors were attached that was worn by the country’s top-level horse riding expert. Based on this database, an analysis of the rider’s practical movements is performed by calculating the elbow angle and the location of the hip. The calculated hip motion value is used to perform the classification of the horse’s gaits through the transformation by a WP. The experimental results obtained by the FCM-NFC are compared with those of the previous classification algorithms, such as a neural network classifier (NNC), naive Bayesian classifier (NBC), and radial basis function network classifier (RBFNC).

In Section 2, the construction of an equestrian motion database, including motion analyzing techniques, is described. In Section 3, the classification algorithms for the original data and the data transformed by a WP are compared. Section 4 presents the study conducted on the characterization of horses’ gaits and classification, as well as the results. Finally, Section 5 contains the conclusion of this paper.

## 2. Construction of Horse Rider’s Motion Database

### 2.1. Building the Horse Riding Motion Database

#### 2.1.1. Motion Capture

**Figure 1:**
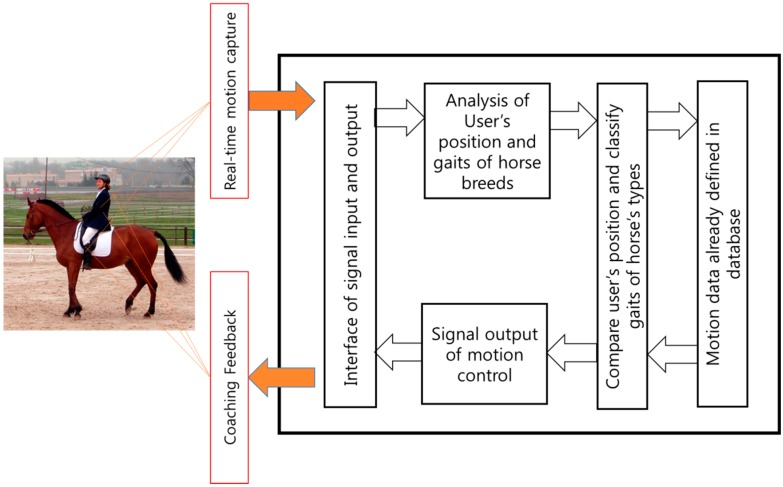
Flow chart of horse rider’s motion capture system.

Motion capture refers to recording human movements in digital form by attaching a sensor to the body or using infrared rays. We used a wireless sensor network manufactured by Xsens Inc (Enschede, The Netherlands) for the motion capture system in horse riding environments. The inertial sensor used for constructing the database is a small and light 9 DOF (depth of field) human orientation tracker that provides drift-free kinematic data. This tracker consists of a three-axis acceleration meter, three-axis gyroscope, and three-axis geomagnetic sensor. The Xsens’ inertial sensor portfolio provides full-body, wearable motion capture solutions. To capture expert’s motion, the rider wears a suit including inertial sensors based on wireless inertial sensors. The motion data are transmitted to a computer, which then compares the data. The suit is characterized by allowing calibration, real-time capture screen viewing, simultaneous measurement, and previously measured motion data readings. Figure 1 shows a flow chart of the rider’s motion capture system.

**Figure 2:**
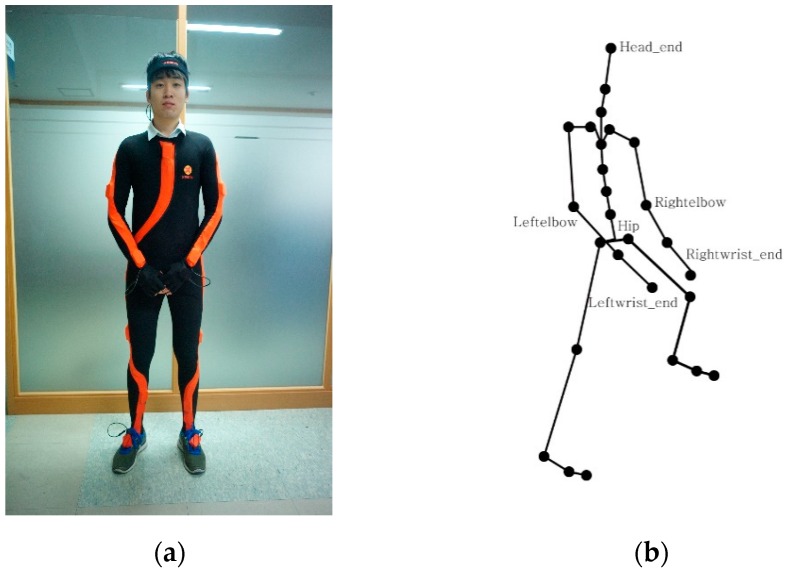
(**a**) Motion capture suit consisting of 16 inertial sensors; (**b**) BVH human structure chart.

Data received through the motion capture system can be exported into BVH (bounding volume hierarchy) files. The BVH files can be stored in 3D file format using the open software BVHViewer. The 3D file contains the coordinate at every measurement site, stored according to frame. Twenty-eight human measurement body sites are displayed as 28 points, as shown in Figure 2. In the data arrangement, lines form a frame and 84 rows represent the locations (*x*, *y*, and *z*) of the three axes for the 28 sites.

The 28 sites consist of hips, breast, breast 2, breast 3, breast 4, neck, head, head end, right nape, right shoulder, right elbow, right wrist, right wrist end, left nape, left shoulder, left elbow, left wrist, left wrist end, right hip, right knee, right ankle, right toe, right tiptoe, left hip, left knee, left ankle, left toe, and left tiptoe. Figure 2a shows a man wearing a suit to which 16 inertial sensors are attached and Figure 2b shows the human structure chart obtained by BVH software.

#### 2.1.2. Database Construction in Horse Riding Environment

**Figure 3:**
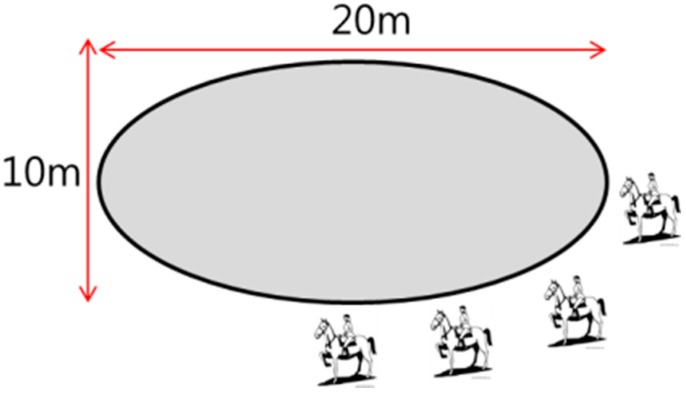
Database construction environment.

Motions were acquired from a horse riding expert who made one or two revolutions per gait (walk, sitting trot, rising trot, canter) of an oval horse riding course 20 m in length and 10 m in breadth while wearing a motion capture suit. Figure 3 shows the database construction environment. 

**Figure 4:**
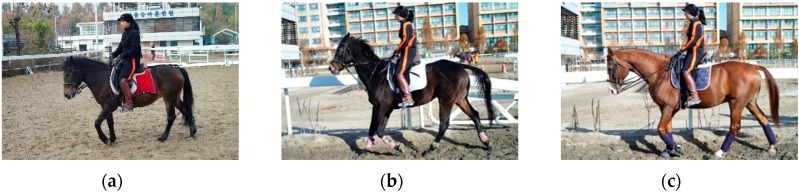
Three breeds of horse: (**a**) Jeju; (**b**) Thoroughbred; (**c**) Warmblood.

The expert, whose career is in national athletics, is female, 164 cm in height, and 235 mm in foot size. Using the 3D motion capture suit based on Xsens inertial sensors, data were extracted in the order of Jeju (137 cm or less), Thoroughbred (160 cm), and Warm Blood (150–173 cm). It took 1 to 2 min to measure a file. Fifteen data were received per gait. A horse’s gaits consist of walk, sitting trot, rising trot, and canter. In the walk gait, the horse moves at 130 m a minute, approximately 8 kph; in the sitting trot gait at 220 m per minute, approximately 13 kph; in the canter gait at 350 m per minute, approximately 21 kph; and at full gallop 100 m per minute, that is 60 kph; the maximum speed is 72 kph. The test used a total of four gaits: walk, sitting trot, rising trot, and canter. The measured frame rate was 100 frames/s (fps). Figure 4a–c shows the three breeds of horse, Jeju, Thoroughbred, and Warmblood, respectively.

#### 2.1.3. Gait-Specific Motions in Real Horse Riding Environment

**Figure 5:**
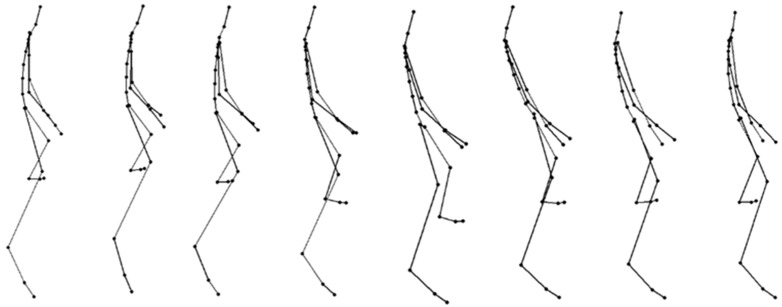
Motion data for the canter gait in BVH environment.

The cycles of gait-specific professional motions were presented in the order of frames using the BVH motion analysis program. Figure 5 visualizes canter motion data at specific frame intervals between 10 and 15 frames.

### 2.2. Method for Analyzing Real Horse Riding Postures

To achieve the correct posture while horse riding, the user’s motions must be analyzed. A comparative analysis is performed by using the following two methods (elbow angle and hip (*y*)).

#### 2.2.1. Elbow Angle

**Figure 6:**
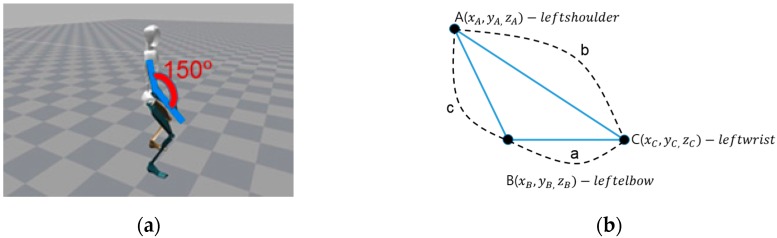
Visualization of elbow angle. (**a**) Elbow angle visualization in MVN studio software; (**b**) Geometric representation for elbow calculation.

Three elbow coordinates, A, B, and C, are defined using Equation (1) by extracting the values of the body feature points A (shoulder), B (elbow), and C (wrist) from a sensor. Figure 6 visualizes a method for calculating the elbow angle using MVN studio motion capture software.

(1) $$A=x_{A},y_{A},z_{A},\;B=x_{B},y_{B},z_{B},\;C=x_{C},y_{C},z_{C}$$

The distance between feature points A (wrist), B (elbow), and C (shoulder) can be calculated using by

(2) $$\bar{AB}=\sqrt{{\left(x_{A}-x_{B}\right)}^{2}+{\left(y_{A}-y_{B}\right)}^{2}+{\left(z_{A}-z_{B}\right)}^{2})}=c\;\bar{BC}=\sqrt{{\left(x_{B}-x_{C}\right)}^{2}+{\left(y_{B}-y_{C}\right)}^{2}+{\left(z_{B}-z_{C}\right)}^{2})}=a\;\bar{CA}=\sqrt{{\left(x_{C}-x_{A}\right)}^{2}+{\left(y_{C}-y_{A}\right)}^{2}+{\left(z_{C}-z_{A}\right)}^{2})}=b$$

Equation (3) is entered, if a distance is calculated for each feature point:

(3) $$b^{2}=c^{2}+a^{2}-2ca\cos B\;c^{2}=a^{2}+b^{2}-2ab\cos C$$

It is possible to calculate the angle of an elbow joint, if a transformation is made, as

(4) $$\text{Elbow angle}=\cos^{-1}\left(\frac{c^{2}+a^{2}-b^{2}}{2ca}\right)$$

#### 2.2.2. $Hip_{y}$ Location

**Figure 7:**
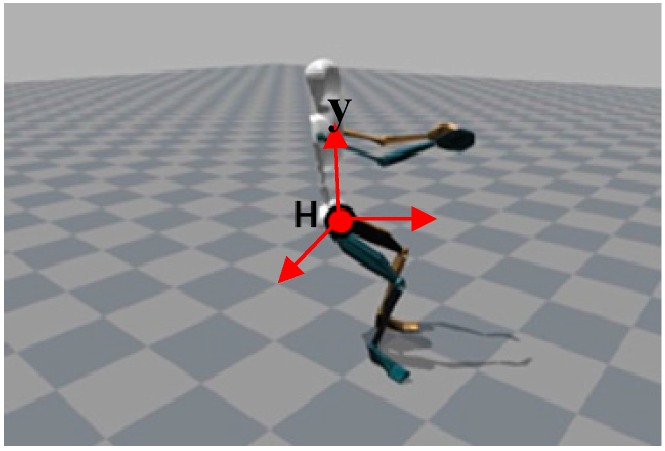
Visualization of hip value.

A coordinate H (*x*,*y*,*z*) is obtained by extracting the hip values from the database collected by inertial sensors. Figure 7 shows the visualization of the hip value in MVN studio motion capture software. These hip motion data are used to classify the horse’s gaits in the design of the classifier. The $Hip_{y}$ is *y*-axis (vertical axis) component of hip position. These values represent the rhythm of rider motion according to horse gaits.

(5) $$Hip_{y}=H_{\text{value of vertical axis}}$$

### 2.3. Horse Simulator and Riding Coaching System

**Figure 8:**
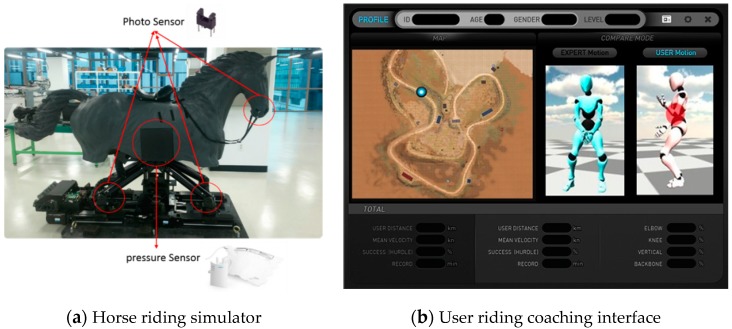
Horse riding simulator and user interface for riding coaching.

We developed a 5-senses convergence sports simulator as a horse riding simulator based on a multi-axis motion platform, as shown in Figure 8a. The coaching system using the classification of horse gaits can be applied to this simulator. The horse simulator is equipped with 26 photo sensors and two pressure sensors to obtain information from the simulator as you can see Figure 8. A photoelectric sensor, or photo eye, is used to discover the distance, absence, or presence of an object by using a light transmitter, frequently infrared, and a photoelectric receiver. A pressure sensor measures pressure, typically of gases or liquids. Pressure is an expression of the force required to stop a fluid from expanding, and is usually stated in terms of force per unit area. Figure 8b shows the graphical user interface for riding coaching in real-time and off-line environments. In the same manner as for real riding, a database was constructed from data collected from a motion capture suit to which 16 inertial sensors were attached worn by the country’s top-level horse riding expert. As shown in Figure 8b, the coaching system compares the expert’s motion with the user’s motion and informs the user of the correct riding motion corresponding to the classified riding gait through text and speech on the basis of the motion analysis as mentioned above [30].

## 3. Machine Learning Algorithms

In this section, we address a WP for transforming and compressing the original sensor data. Further, we used NNC, RBFNC, NBC, and FCM-NFC to predict the horse’s gaits for both real-time and off-line riding coaching.

### 3.1. Dimension Reduction Algorithm

#### 3.1.1. Wavelet

**Figure 9:**
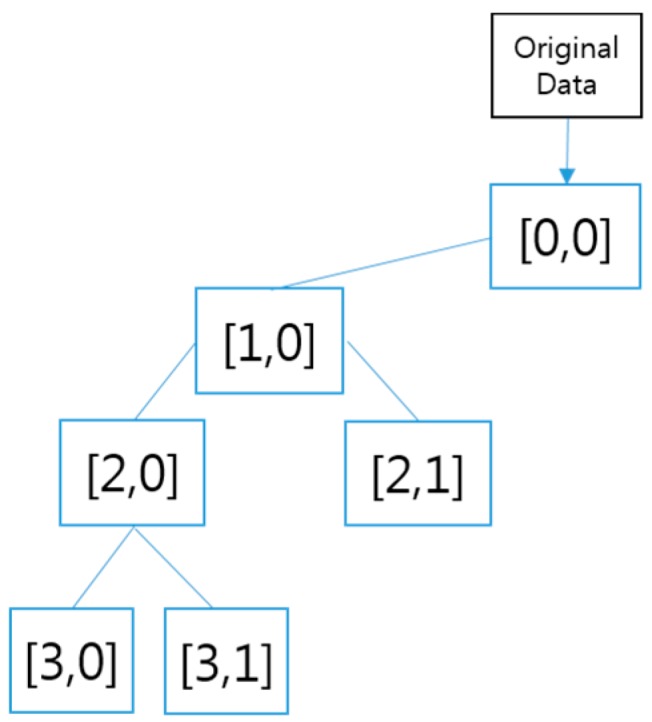
Partial wavelet decomposition structure.

A wavelet is a wave-like vibration, where the breadth of the vibration repeatedly increases and decreases, with a focus on 0. It emerges in the typical form of a “short vibration,” as recorded in a seismograph or electrocardiogram graph. In general, a wavelet is exploited for treating signals. It can be used to extract information from an unknown source by combination with a known source using a convolution technique. A wavelet is a mathematical tool that can be used to extract not only audio signals and images, but also various kinds of data. A series of wavelets is additionally needed to analyze data completely. Such “complementary” wavelets can decompose data without leading to a difference in the data or overlapping. Therefore, the decomposition process is mathematically reversible. Therefore, wavelets are useful in wavelet-based compression/release algorithms designed to minimize loss and restore original information. Mathematically, this expression technique constitutes a set of complete orthogonal basis functions for the Hilbert space of square-integrable functions, an overcomplete set, or a set of square-integrable functions on a vector space frame. Figure 9 shows a wavelet decomposition structure that performs dimension reduction to provide time saving and precision. Here, the input data are the horse gait data (100 × 160) obtained by building a horse rider’s *y*-axis data according to the horse’s gait. Dimension reduction allows data of Layer 0 [0,0], Layer 1 [1,0], Layer 2 [2,0], and Layer 3 [3,0] (13 × 160) to be extracted. The size of Layers 0, 1, and 2 is 100 × 60, 50 × 150, and 25 × 160, respectively.

#### 3.1.2. Wavelet *vs.* Wavelet Packet

**Figure 10:**
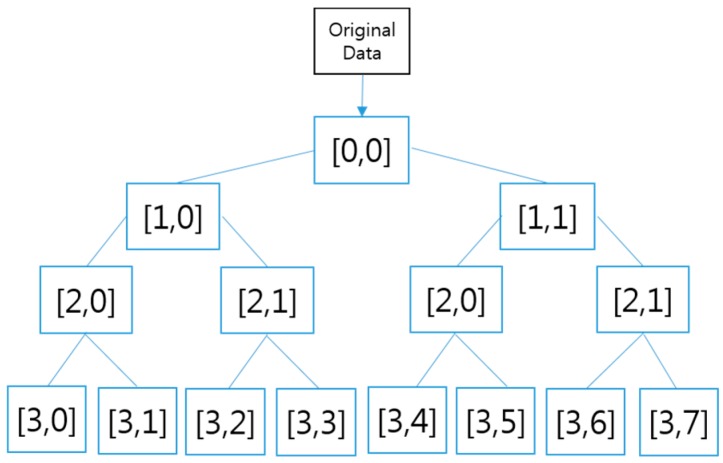
Decomposition structure of wavelet packet.

In a wavelet, decomposition continues to occur only in low frequency components after the low and high frequency components from the first data are decomposed, as shown in Figure 9. In contrast, in a WP decomposition occurs regardless of a low or high frequency and level decomposed in $2^{n}$. Here n is levels of decomposition. For n levels of decomposition the wavelet packet decomposition (WPD) produces $2^{n}$ different sets of coefficients. However, due to the down-sampling process the overall number of coefficients is still the same and there is no redundancy. Figure 10 shows the decomposition steps of a WP. The input data are horse data (100 × 160), the same as the data used for the above wavelet. Dimension reduction allows data of Layer 0 [0,0], Layer 1 [1,0], Layer 2 [2,0], and Layer 3 [3,0] to be extracted. The size of Layers 0, 1, 2, and 3 is 100 × 160, 50 × 160, 25 × 160, and 13 × 160, respectively. It can be seen that decomposition is performed at high frequency, unlike in a wavelet. An excellent classification rate is achieved by executing all the WP feature data from Layer 0 to Layer 3.

The wavelet packet is a generalization form of wavelet decomposition that performs signal analysis. This method is accomplished by three parameters such as frequency, position and scale as in wavelet decomposition. In the procedure of wavelet decomposition, the first step splits the approximation coefficients into two parts. After splitting we obtain a vector of approximation coefficients and detail coefficients, respectively. The information lost between two successive approximations is captured in the detail coefficients. The next step consists in splitting the new approximation coefficient vector. In the corresponding wavelet packets situation, each detail coefficient vector is also decomposed into two parts using the same approach as in approximation vector splitting [31,32].

### 3.2. Classifier Algorithms

#### 3.2.1. Neural Network Classifier

**Figure 11:**
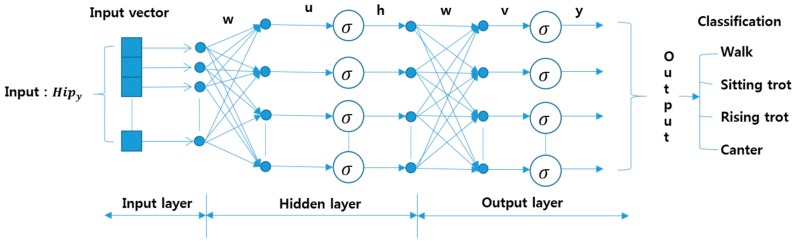
Architecture of neural network classifier (NNC).

The neural network is a structure adopted in computer programs to solve problems in a similar way to human brain processing. In other words, when neurons, that is, nodes or connection points, form a network by mutual connection, the network is called a neural network [26,27]. Figure 11 shows the basic structure of a neural network. The horse gait data consisted of a rider’s *y*-axis data (100 × 160) and WP feature data (25 × 160). Since we used 50% of the data as the input, the size of the hip data for the walk gait was 100 × 20. The input vector for training classifier consists of vector including *y*-axis component of hip position. These values represent the rhythm of rider motion according to horse gaits. We use original data points and the data transformed by wavelet packet in this paper. The angle of elbow, knee, backbone, and distance of each elbow were used for motion analysis and coaching [33,34].

#### 3.2.2. Naive Bayesian Classifier

Naive Bayes is a stochastic classifier and a model that hypothesizes that all features are conditionally independent, if class variables are given. The Bayesian network shows structures are independent, if class variables are given. The naive Bayesian classifier is very efficient in terms of learning and application. The parameters composing a model are limited to those for probability distribution. A learned model can be also applied efficiently. The naive Bayesian classifier exercises an optimum performance, if it meets a conditional independent hypothesis with probability distribution. The performance of the naïve Bayesian classifier has been proved experimentally and theoretically. However, many current problems do not follow the naive Bayesian hypothesis. Specifically, there are many problems in which the specific variables are not conditionally independent. The performance is expected to be degraded, if each specific variable is not conditionally independent. If each variable has a binary value, the expressiveness of the naive Bayesian classifier is the same as that of a linear classifier [35,36].

#### 3.2.3. Radial Basis Function Network Classifier

**Figure 12:**
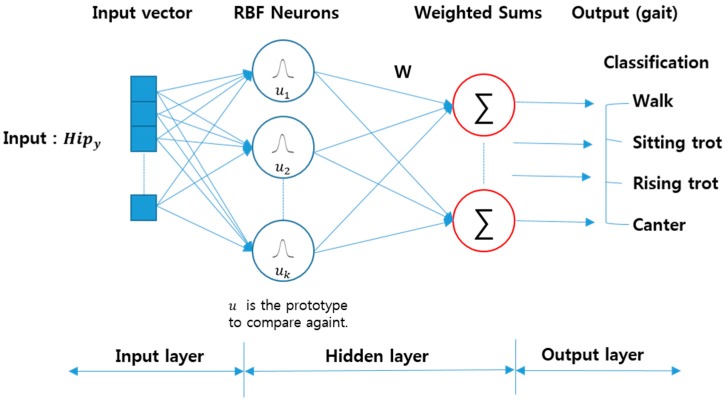
Architecture of the radial basis function network classifier (RBFNC).

In the field of mathematical modeling, the radial basis function network classifier (RBFNC) is an artificial neural network and uses radial basis functions as sigmoid functions. The output of the network is a linear combination of the radial basis functions of the input and neuron parameters. The RBFN is used for function approaches, time series prediction, classification, system control, *etc.* Figure 12 illustrates the RBFNC’s architecture [37]. The horse gait data consisted of a horse rider’s *y* data (100 × 160) and WP feature data (25 × 160). Since we used 50% of the data as the input, the size of the hip data in the walk gait was 100 × 20.

#### 3.2.4. FCM-Based Neuro-Fuzzy Classifier (NFC)

**Figure 13:**
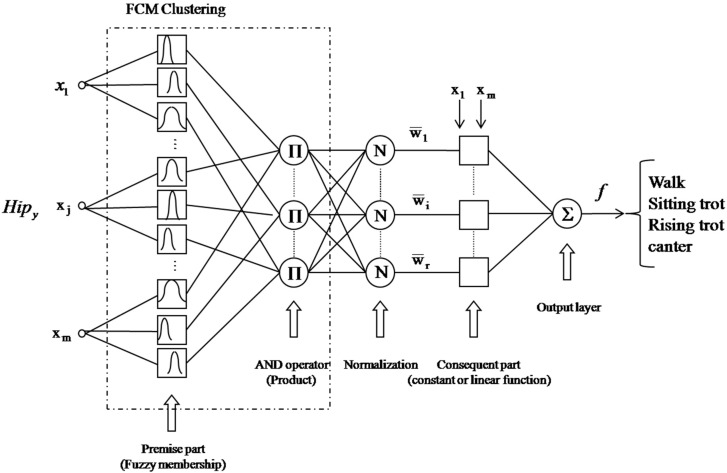
Architecture of NFC based on FCM clustering (FCM-NFC).

The design of the FCM-NFC consists of an NFC assisted by FCM clustering. Here, the NFC is similar to the adaptive neural fuzzy inference system (ANFIS) introduced by Jang [38]. While Jang’s model frequently encounters the “curse of dimensionality” problem that the number of fuzzy rules exponentially increases because of the grid partitioning of the input space, the FCM-NFC can solve such a problem by virtue of the flexible scatter partitioning of FCM clustering. In general, a fuzzy classifier has an appropriate reasoning ability that is easy to apply to a complicated or non-linear system using professional and experiential knowledge and can overcome the vagueness or uncertainty inherent in the human thinking process. However, professional knowledge is often inconsistent and sometimes incomplete. There are also difficulties in acquiring fuzzy rules by human intuition and experience due to the lack of a systematic and efficient method. To confront this problem, it is frequently advantageous to use several computing techniques synergistically rather than exclusively, resulting in the construction of complementary hybrid intelligent systems. Thus, we attempted to combine the fuzzy system with a neural network. Figure 13 shows the architecture of the FCM-NFC. The classifier shown in s has an inference system with two TSK-type fuzzy rules as follows [38].

Rule 1: If *x_1_* is *A_1_* and … x*_m_* is *B_1_*, and then *f* is *f_1_* Rule n: If *x_1_* is *A_n_* and … x*_m_* is *B_n_*, and then *f* is *f_2_* (6)

where *f_i_* is the linear equation of *i*’th consequent part. The linguistic labels in the first layer are constructed by Gaussian membership functions with two parameters as

(7) $$A_{j}^{i}(x_{j})=\exp\{-{\left(\frac{x_{j}-c_{j}^{i}}{\sigma_{j}}\right)}^{2}\}$$

Each of the cluster centers generated by FCM clustering represents a prototype that exhibits certain characteristics of the system to be modeled. The final inference output of the FCM-NFC is computed as the weighted average method

(8) $$f=\sum_{i=1}^{r}{\bar{w}}_{i}f_{i}=\frac{\sum_{i=1}^{r}w_{i}f_{i}}{\sum_{i=1}^{r}w_{i}}$$

where ${\bar{w}}_{i}$ is a normalized firing strength of the *i*’th rule. These values are obtained by the ratio of the *i*’th rule’s firing strength to the sum of all rule’s firing strengths. The learning scheme of the proposed FCM-NFC is realized by hybrid learning method using a back-propagation (BP) algorithm and least square estimator (LSE). Fuzzy c-means (FCM) clustering is a method of clustering that allows one data point to belong to two or more clusters. This method is frequently used in pattern recognition. It is based on the minimization of the objective function

(9) $$J_{m}=\overset{N}{\underset{i=1}{\sum }}\overset{N}{\underset{i=1}{\sum }}{u^{m}}_{ij}{∥x_{i}-c_{j}∥}^{2}\;,\;1\leq m<\infty$$

where *m* is any real number greater than 1, $u_{ij}$ is the degree of membership of $x_{i}$ in the cluster *j*, $x_{i}$ is the *i’*th piece of d-dimensional measured data, $c_{j}$ is the d-dimension center of the cluster, and ∥·∥ is any norm expressing the similarity between any measured data and the center. Fuzzy partitioning is performed through an iterative optimization of the objective function shown above, with the update of membership $u_{ij}$ and the cluster centers $c_{j}$ by

(10) $$u_{ij}=\frac{1}{\sum_{k=1}^{C}{\left(\frac{∥x_{i}-c_{i}∥}{∥x_{i}-c_{k}∥}\right)}^{\frac{2}{m-1}}}$$

(11) $$c_{j}=\frac{\sum_{i=1}^{N}{u^{m}}_{ij}\cdot x_{i}}{\sum_{i=1}^{N}{u^{m}}_{ij}}$$

This iteration terminates when $\max_{ij}\left\{\left|{u^{\left(k+1\right)}}_{ij}-{u^{\left(k\right)}}_{ij}\right|\right\}$ < ε, where ε represents a termination criterion between 0 and 1, whereas *k* represents the iteration steps. This procedure converges to a local minimum or a saddle point of $J_{m}$. The algorithm is composed of the following steps:

[Step 1]  Initialize U = [$u_{ij}$] matrix, $U^{\left(o\right)}$
[Step 2]  At k-step : calculate the centers vectors $C^{\left(k\right)}=\left[c_{j}\right]\;\text{with}\;U^{\left(k\right)}$
[Step 3]  Update $U^{\left(k\right)},U^{\left(k+1\right)}$
[Step 4]  If $∥U^{\left(k+1\right)}-U^{\left(k\right)}∥<\varepsilon$, then stop; otherwise, retun to Step 2.

## 4. Experiment and Results

### 4.1. Horse Rider’s Motion Database by Riding Gaits

In this section, we describe the construction of a horse rider’s motion database for four horse gaits of three breeds of horse (Jeju, Warmblood, and Thoroughbred). The data in this database were obtained from a motion capture suit including inertial sensors worn by a horse riding expert. From among several data, we used the hip values of the *y*-axis for horse gait classification. To synchronize this database, the minimum value was extracted between 1 and 400 frames and 100 values were extracted from the point of time one. In order to achieve a standard performance, all the experiments were completed in the 10-fold cross-validation mode. The entire data set used in this study comprised 80 data. The training and validation data set were randomly selected by a 50%/50% split, respectively. The training data set was used for predictor construction, while the test data set was used for predictor validation. Thus, the resultant predictor was not biased toward the training data set and it was likely to have a better generalization capacity to new data.

#### 4.1.1. Horse Riding Learning Data and Validation Data

**Figure 14:**
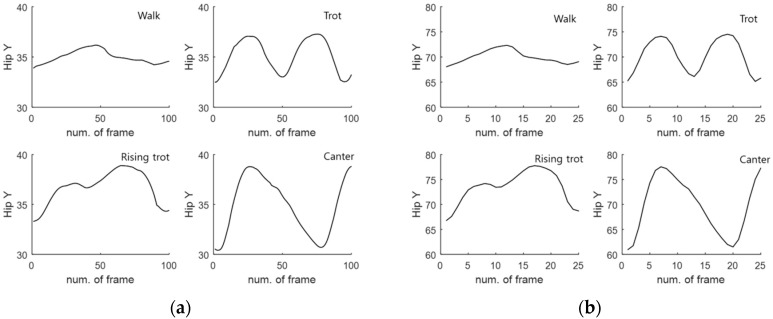
Hip motion data (**a**) original sensor data (walk, sitting trot, rising trot, canter) (**b**) data transformed by wavelet packet (walk, sitting trot, rising trot, canter)

**Figure 15:**
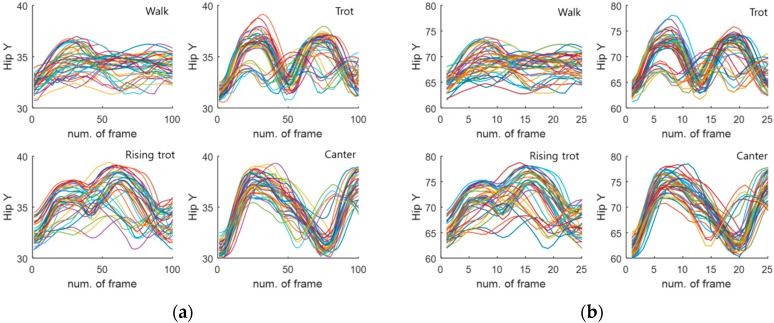
Overlapped hip motion data. (**a**) original sensor data (walk, sitting trot, rising trot, canter) (**b**) data transformed by wavelet packet (walk, sitting trot, rising trot, canter).

Figure 14 shows some of the hip motion data for four horse gaits (walk, sitting trot, rising trot, canter). As shown in Figure 14, the hip motion for each gait has unique characteristics. In the case of the walk gait, we can see that the motion is flat. In the case of the sitting trot gait, the motion shows an iterative curve, because the trot is a two-beat diagonal gait of the horse, where the diagonal pairs of legs move forward at the same time with a moment of suspension between each beat. Figure 15 visualizes several overlapped hip motion data. Table 1 lists the database information for the four gaits. The size of this database is 100 × 160. Here, the number of dimensions is 100. We divided it into the validation data with a size of 100 × 80 and the learning data with a size of 100 × 80.

The initial center of FCM is randomly generated by the membership matrix U with random values between 0 and 1 such that the summation of degrees of belongingness for a data set always is equal to unity. We selected 33 if-then rules through trial and error as the number of rule increases between 2 and 50. The size of cluster centers after performing FCM clustering is 33 × 25. The size of membership matrix is 33 × 80 for training and testing data, respectively. The output is class number representing horse gaits to be classified. The size of output is also 80 × 1. The input vector for training classifier consists of vector including *y*-axis component of hip position. These values represent the rhythm of rider motion according to horse gaits. We use original data points and the data transformed by wavelet packet in this paper. The angle of elbow, knee, backbone, and distance of each elbow were used for motion analysis and coaching.

#### 4.1.2. Features Transformed by Wavelet Packet

**Figure 16:**
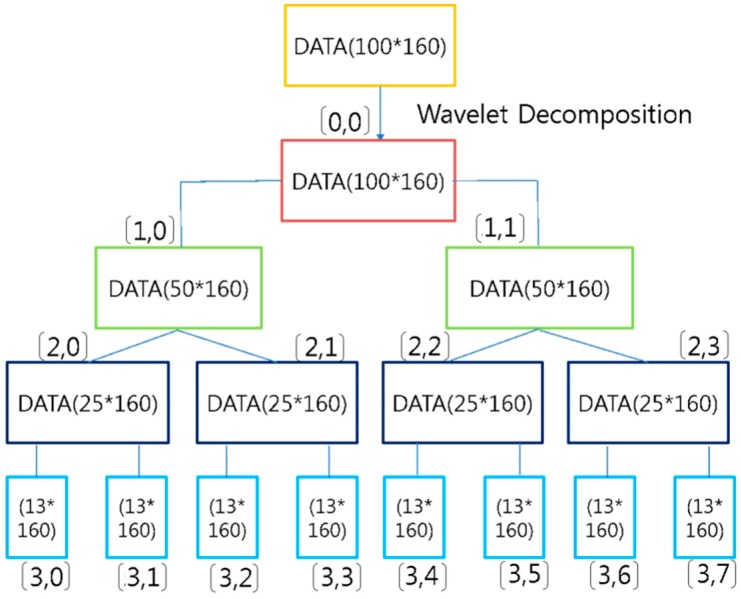
Wavelet packet decomposition.

The features are extracted by applying the training data and the validation data based on a WP. The transformed data sets consist of four layers. Each layer is composed of [0,0], [1,0], [1,1], [2,0], [2,1], [2,2], [2,3], [3,0], [3,1] [3,2], [3,3], [3,4], [3,5], [3,6], and [3,7], as shown in Figure 16. Figure 16 shows the decomposition step of a WP consisting of four layers. It is possible to generate a total of 14 feature data, *i.e.*, 2 in Layer 1, 4 in Layer 2, and 8 in Layer 3. Here, we used the transformed data (25 × 160) of Layer 2 as feature data in consideration of the recognition rate and velocity. Thus, the size (100 × 160) of the original data is transformed into a reduced size (25 × 160) by the WP. We divided this transformed database into validation data with a size of (25 × 80) and learning data with a size of (25 × 80). Table 2 lists the information of the database transformed by the WP. Figure 14 visualizes the hip motion data and transformed by the WP for the four gaits. Figure 15 visualizes the overlapped and transformed motion data and transformed by the WP for the four gaits.

### 4.2. Experimental Results

The experiments were performed using a computer with a 3.4 GHz CPU, Intel (R) Core (TM) i7-2600, 16 Gbyte memory, and MATLAB R2012b. The size of the original data and the transformed data was 100 × 160 and 25 × 160, respectively. The experimental results of the RBFNC showed a classification accuracy performance of 25%, as the learning failed in the case of the original motion data. However, the experimental results showed a classification accuracy performance of 86.25% for the feature data set transformed by the WP. Here, we selected 236 nodes and a learning rate of 0.022 through trial and error in the design of RBFNC.

**Figure 17:**
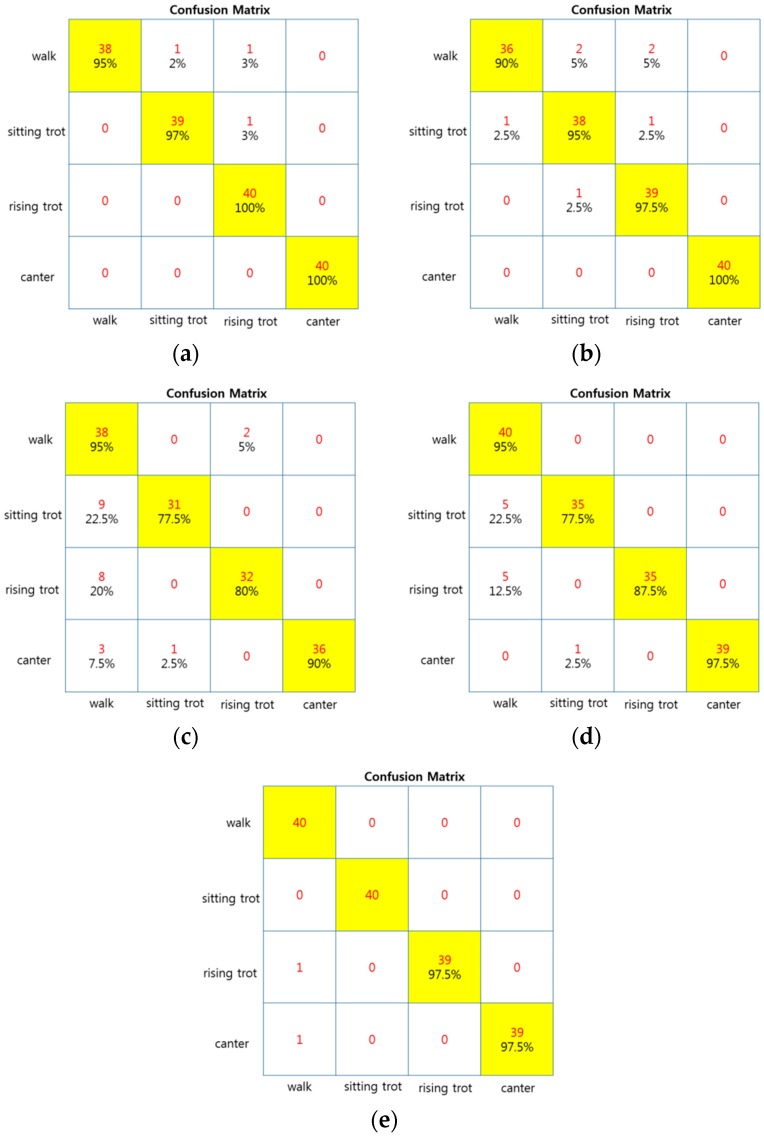
Confusion matrix (**a**) NNC (**b**) SVM (**c**) NBC (**d**) RBFM (**e**) FCM-NFC.

**Figure 18:**
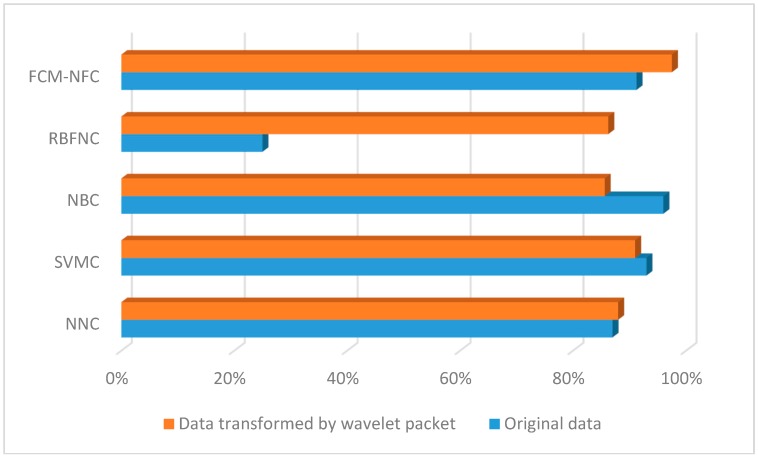
Performance comparison between the FCM-NFC and other classifiers.

In the case of the FCM-NFC, the results showed a classification accuracy performance of 91.25%, when using the original motion data. We used 33 fuzzy if-then rules of the TSK-type by finding the optimal number of rules that showed the minimum error for the validation data set. Furthermore, we obtained the best classification performance, 97.5%, when using the transformed data set as listed in Table 3. Here, we selected 50 rules in the same manner as above. Here, the number of rules is the same as that of cluster centers estimated by FCM clustering. Figure 17 shows confusion matrix of all algorithms(NNC, SVM, NBC, RBFM, FCM-NFC). Figure 18 shows a bar graph visualizing the classification performance. Table 4 lists the processing time of NNC, NBC, RBFNC, and FCM-NFC for the classification of horse gaits.

## 5. Conclusions

In this study, we compared horse riders’ motion features (elbow angle, hip position) and the gaits (walk, sitting trot, rising trot, and canter) of the horse breeds Jeju, Warm Blood, and Thoroughbred in a database consisting of the data collected from a suit with 16 inertial sensors worn by the country’s top-level horse riding expert, using the Euclidean calculation method. The comparison showed that there were differences between the data feature values obtained for the horse and gait types. For gait classification and coaching, the features were extracted using a multiple signal WP and the algorithm’s performance was evaluated when using the NNC, NBC, RBFNC, and FCM-NFC. The NBC showed a classification performance of 96% for the original motion data, and the FCM-NFC showed a 97.5% (the highest) performance for the motion data transformed by the WP. It is concluded that the FCM-NFC has a good classification capacity and is effective. On the basis of the classification results and the motion information such as the angle of elbow, knee, backbone, and distance of each elbow for motion analysis and coaching, we can apply to coaching system by each horse gait for rider under real or horse simulator environments.

## Figures and Tables

**Table 1:** Original data.

	Walk	Sitting Trot	Rising Trot	Canter	Total
Training data	100 × 20	100 × 20	100 × 20	100 × 20	100 × 80
Test data	100 × 20	100 × 20	100 × 20	100 × 20	100 × 80
Total	100 × 40	100 × 40	100 × 40	100 × 40	100 × 160

**Table 2:** Size of the feature data transformed by wavelet packet for training and testing data.

	Walk	Sitting Trot	Rising Trot	Canter	Total
Training data	25 × 20	25 × 20	25 × 20	25 × 20	25 × 80
Testing data	25 × 20	25 × 20	25 × 20	25 × 20	25 × 80
Total	25 × 40	25 × 40	25 × 40	25 × 40	25 × 160

**Table 3:** Performance comparison for original sensor data and the transformed data.

	NNC	SVM	NBC	RBFNC	FCM-NFC
Original data	87%	93%	96%	25%	91.25%
Transformed data by WP	88%	91%	85.62%	86.25%	**97.5%**

**Table 4:** Comparison of processing time (s).

	NNC	NBC	RBFNC	FCM-NFC
Original Data	2.8	0.03	28.26	0.8
Data transformed by WP	2.5	0.028	5.13	0.61
